# Head and Neck Cancer: A Study on the Complex Relationship between QoL and Swallowing Function

**DOI:** 10.3390/curroncol30120753

**Published:** 2023-12-06

**Authors:** Daniel Strüder, Johanna Ebert, Friederike Kalle, Sebastian P. Schraven, Lennart Eichhorst, Robert Mlynski, Wilma Großmann

**Affiliations:** 1 Department of Otorhinolaryngology, Head and Neck Surgery “Otto Körner”, Rostock University Medical Center, D-18057 Rostock, Germany; johanna.ebert@uni-rostock.de (J.E.); friederike.kalle@med.uni-rostock.de (F.K.); lennart.eichhorst@med.uni-rostock.de (L.E.); robert.mlynski@med.uni-rostock.de (R.M.); wilma.grossmann@med.uni-rostock.de (W.G.); 2 Department of Otorhinolaryngology, Head and Neck Surgery, RWTH Aachen University Hospital, D-52074 Aachen, Germany; spschraven@ukaachen.de

**Keywords:** head and neck squamous cell carcinoma (HNSCC), quality of life (QoL), MD Anderson Dysphagia Inventory (MDADI), post-treatment challenges, swallowing function, fiberoptic endoscopic evaluation of swallowing (FEES), Penetration–Aspiration Scale (PAS), subjective vs. objective assessments, disease severity, therapeutic outcome evaluation

## Abstract

Head and neck squamous cell carcinoma (HNSCC) is linked to significant morbidity, adversely affecting survival and functional capacity. Post-treatment challenges such as pain, dysphonia, and dysphagia are common, prompting increased attention in survivorship research. Quality of Life (QoL) questionnaires, especially the MD Anderson Dysphagia Inventory (MDADI), are prevalent outcome measures in clinical studies but often lack parallel objective swallowing function evaluations, leading to potential outcome discrepancies. This study aimed to illuminate the relationship between subjective QoL (EQ-5D-5L and MDADI) measures and objective swallowing function (evaluated via Fiberoptic Endoscopic Evaluation of Swallowing, FEES) in patients with HNSCC. The analysis revealed a notable discordance between objective measures of swallowing function, such as the Penetration–Aspiration Scale (PAS) and residue ratings in the vallecula or piriform sinus, and patients’ subjective QoL assessments (*p* = 0.21). Despite the lack of correlation, swallowing-related QoL, as measured by the MDADI, was more indicative of disease severity than generic QoL assessments. Generic QoL scores did not demonstrate substantial variation between patients. In contrast, MDADI scores significantly declined with advancing tumor stage, multimodal therapy, and reliance on feeding tubes. However, the clinical significance of this finding was tempered by the less than 10-point difference in MDADI scores. The findings of this study underline the limitations of QoL measures as standalone assessments in patients with HNSCC, given their reliance on patient-perceived impairment. While subjective QoL is a crucial aspect of evaluating therapeutic success and patient-centric outcomes, it may fail to capture critical clinical details such as silent aspirations. Consequently, QoL assessments should be augmented by objective evaluations of swallowing function in clinical research and practice to ensure a holistic understanding of patient well-being and treatment impact.

## 1. Introduction

Head and neck squamous cell cancer (HNSCC) is the 7th most common cancer worldwide and is associated with a poor outcome [1,2,3,4]. Despite aggressive surgery, radiation, and chemotherapy, ~50% of patients die, while survivors suffer from pain, dysphonia, and dysphagia [5].

Among these functional impairments, dysphagia assumes particular significance. Notably, 45–88% of patients endure dysphagia with pharyngeal residue, laryngeal penetration and aspiration. A total of 70% of aspirations occur silently, escaping patient notice [6,7,8,9,10]. Consequently, dysphagia leads to malnutrition, aspiration, and tracheostomies. Given the comprehensive management of HNSCC, a standardized dysphagia diagnosis is imperative. Objective swallow assessments via Fiberoptic Endoscopic Evaluation of Swallowing (FEES) and videofluoroscopy (VFSS) offer direct visualization of the swallowing process, analysis of anatomical structure movements, anomaly detection, and support for treatment planning. Owing to dysphagia’s high importance, swallowing function is increasingly employed as an endpoint in clinical studies. The Orator Study notably introduced swallowing function as a primary endpoint in a prospective randomized clinical trial, comparing primarily surgical and radiotherapeutic therapies [11,12]. Swallowing function was assessed using the MDADI questionnaire, which determines swallowing-related quality of life rather than an objective swallowing assessment. Likewise, 70% of studies on dysphagia in HNSCC solely rely on patient-reported outcomes (PRO) [13].

However, the validity of PROs in evaluating swallowing function in HNSCC is controversial: Some studies indicate significant correlations between subjective swallowing difficulties and objective parameters such as oropharyngeal swallowing efficiency, bolus transport time, residue, and aspiration [14,15,16,17]. Nevertheless, most research suggests a tenuous relationship between these measures, particularly concerning penetration and aspiration [18,19,20,21,22,23,24,25,26,27,28,29]. The present study aimed to determine if central European patients with tumors primarily attributable to smoking and/or alcohol can accurately assess their physiologic swallowing functioning using the most widely employed questionnaire, MDADI [12,30,31,32]. Therefore, this study explored the association of generic and swallowing-related QoL with patient characteristics such as tumor stage, therapy, nutritional mode, and tumor localization. Likewise, generic QoL (EQ-5D-5L) and swallowing-related QoL (MDADI) were correlated with objective swallowing function (FEES).

## 2. Materials and Methods

Study Design: A cross-sectional study was conducted to compare the generic QoL, swallowing-related QoL, and objective swallowing function among patients with HNSCC during their cancer aftercare. This study investigated the influence of therapy and patient characteristics on QoL and swallowing function. Additionally, the subjective QoL was correlated with the objective outcomes obtained from the FEES.

Participants: Patients were approached during cancer aftercare at a tertiary university hospital with a certified head and neck cancer center. Written informed consent was obtained from all participants following the local ethics committee (A 2021-0024) according to the 1964 Declaration of Helsinki. Only patients with histologically confirmed head and neck squamous cell carcinoma were included. Eligibility for the study was open to consecutive patients of any age, gender, and treatment modality. Due to the cross-sectional study design, these data (questionnaires and swallowing assessments) were collected at a single time point, without subsequent follow-up.

Assessment Protocol: The German adaptation of the EQ-5D-5L questionnaire was used to evaluate general health-related QoL comprehensively. This questionnaire is designed to explore a spectrum of life domains, specifically mobility, self-care, the performance of usual activities, the experience of pain or discomfort, and the presence of anxiety or depression. Each dimension is individually scored, culminating in an aggregated index value representing the patient’s overall health status. A higher index value indicates a superior QoL. Simultaneously, the study incorporated the German MDADI [33]. The MDADI is an in-depth questionnaire divided into four subscales, each examining a different facet of the impact of dysphagia. The ‘Global’ subscale provides an immediate personal perception of the impact of dysphagia, while the ‘Emotional’ subscale addresses the psychological effects. The ‘Functional’ subscale considers how dysphagia affects daily activities, and the ‘Physical’ subscale measures the somatic aspects of swallowing difficulties. Together, these subscales converge to form a total score that ranges from 20, signaling severe dysphagia-related QoL impairment, to 100, which reflects no such impairment. Higher scores on this scale denote a less affected swallowing-related QoL, providing a detailed understanding of the physical and emotional well-being of the patients in the context of their swallowing abilities [33,34]. Questionnaires were administered digitally or on paper via Evasys Survey Software, version 8.0 (Evasys GmbH, Lüneburg, Germany). Paper-based questionnaires were printed on DIN A4 sheets and distributed on clipboards. Digital questionnaires were presented on 12.9” tablets (Apple iPad Pro, Cupertino, CA, USA) without a stylus, allowing responses to be marked using finger touch. All questions were displayed in a format consistent with the DIN A4 layout. The decision to use digital or paper-based questionnaires was made randomly, depending on the day of assessment.

FEES was performed to assess swallowing function. Consecutive patients assigned to phoniatrics were recruited. All patients with head and neck tumors were eligible. The procedure was conducted in the phoniatrics department by one experienced and trained laryngologist according to a standardized protocol: After an anatomical and sensory-motor assessment, a distal-chip endoscope was used for endoscopic evaluation of swallowing during the administration of three standard consistencies (International Dysphagia Diet Standardization Initiative [IDDSI] consistency, starting at 7 and then transitioning to 0): pureed solids (apple puree/plain yogurt; IDDSI consistency, 4), thin liquids (water; IDDSI consistency, 0), and solids (cracker; IDDSI consistency, 7) in successive boluses that increased in size, starting with a level teaspoon amount (puree/water) or small sip followed by larger boluses (heaped teaspoon puree/several sips of water out of medicinal cup). At least three teaspoon amounts were administered to evaluate spilling, residue, penetration, and aspiration. If aspiration occurred, the examiner decided if it was safe to administer another bolus of the same size and viscosity to determine whether the patient still demonstrated aspiration-otherwise the remaining boluses of this consistency were skipped, and the smallest amount of the next consistency was administered. In cases of gross aspiration, the examination was aborted. Food coloring was used to enhance contrast, and all FEES examinations were video and sound-recorded for later assessment. Swallowing efficacy and safety were quantified utilizing two scales: The Penetration–Aspiration Scale (PAS), an eight-point scale developed by Rosenbek, was employed to characterize the depth of bolus misdirection and the patient’s response to it [35,36]. The highest PAS score achieved by a participant, indicative of the greatest impairment across all bolus consistencies, was recorded for subsequent analysis. Additionally, the Yale Pharyngeal Residue Severity Rating Scale (YPRSRS), a five-point scale, was used to evaluate the presence and severity of pharyngeal residue in the vallecula and piriform sinus [37]. The FEES and subsequent scoring were conducted in accordance with validated guidelines to ensure the fidelity and consistency of the evaluation process [38]. 

Statistics: Statistical analysis was conducted using GraphPad PRISM software, version 9 (GraphPad, San Diego, CA, USA). Quantitative variables were presented as means (±standard deviations) and medians (including ranges min. to max.). Descriptive statistics were used to summarize these data, and the D’Agostino–Pearson normality test was performed to assess normal distribution. T-tests were employed to compare two independent parametric samples. The ordinary one-way ANOVA with Tukey’s T3 posthoc test was used for multiple comparisons. In the case of unequal variances, Welch’s ANOVA, followed by Dunnett’s T3 posthoc test, was conducted. For non-parametric data, the Mann–Whitney U test was applied. Non-parametric multiple comparisons were performed using the Kruskal–Wallis and Dunn’s multiple comparisons tests. Spearman’s non-parametric correlation was used to calculate correlations between individual parameters, and two-tailed *p*-values were reported. Statistical significance was defined as *p* < 0.05.

## 3. Results

### 3.1. Characteristics of the Study Cohort

This study enrolled 307 patients, with a significantly higher proportion of men (79.81%, *n* = 245) than women (20.33%, *n* = 62) (Table 1). The mean age of the participants was 65.88 years, with a standard deviation of 9.98 years. The most frequently affected sites were the oropharynx (40.39%, *n* = 124) and the larynx (32.25%, *n* = 99). The distribution of primary tumor sizes was as follows: 29.97% (*n* = 92) of patients presented with T1 tumors, 26.38% (*n* = 81) with T2, and an equal prevalence of 18.24% for both T3 and T4 tumors (*n* = 56 for each category). The assessment of lymph node involvement (N-Stage) revealed that 44.95% (*n* = 138) of patients had no lymph node metastasis (N0), 15.31% (*n* = 47) were classified as N1, 35.83% (*n* = 110) as N2a/b/c, and a smaller subset of 3.58% (*n* = 11) as N3a/b. Regarding distant metastases (M-Stage), a vast majority of patients were classified as M0, with 91.53% (*n* = 281) showing no distant metastasis, while only 0.98% (*n* = 3) were M1 and 7.49% (*n* = 23) were denoted as Mx. The treatment modalities varied, with 24.10% (*n* = 74) undergoing only surgery, 18.89% (*n* = 58) having surgery plus radiotherapy, and 19.87% (*n* = 61) receiving surgery plus chemoradiotherapy. 36.72% (*n* = 113) received primary radiotherapy or chemoradiation without surgery. The timing for post-therapy evaluations was: 10.42% (*n* = 32) were evaluated at ≤5 months, 13.36% (*n* = 41) at 6–11 months, 45.93% (*n* = 141) between 1–5 years, and 30.29% (*n* = 93) were evaluated at >5 years after therapy. A subset of the population, representing 15.31% (*n* = 47), depended on feeding tubes for nutrition.

A total of 59 patients completed the EQ-5D-5L, MDADI, and standardized FEES assessments. This subgroup had an average age slightly lower than the broader group at 63.20 years (±9.32), with a male representation of 77.97% (*n* = 46). This subgroup showed a higher presence of advanced tumors, with 60% being classified as T3 or T4. Within the subgroup, the distribution of lymph node involvement (N-Stage) was comparable with the main cohort: 37.29% of patients (*n* = 22) had no lymph node metastasis (N0), while 13.56% (*n* = 8) were classified as N1, 37.29% (*n* = 22) as N2, and 11.86% (*n* = 7) as N3. Likewise, regarding the presence of distant metastases (M-Stage), a significant majority, 93.22% (*n* = 55), had no distant metastasis (M0), with a minimal 1.69% (*n* = 1) showing distant metastasis (M1), and 5.08% (*n* = 3) were categorized as Mx. In the FEES subgroup, dependence on feeding tubes was higher at 55.93% (*n* = 33). Treatment types within this group were nearly evenly split between primary surgery (56.94%, *n* = 33) and prior (chemo)radiation (44.06%, *n* = 26), with a larger percentage evaluated within one year post-therapy (67.8%).

### 3.2. Generic and Swallowing-Related QoL

**Figure 1:**
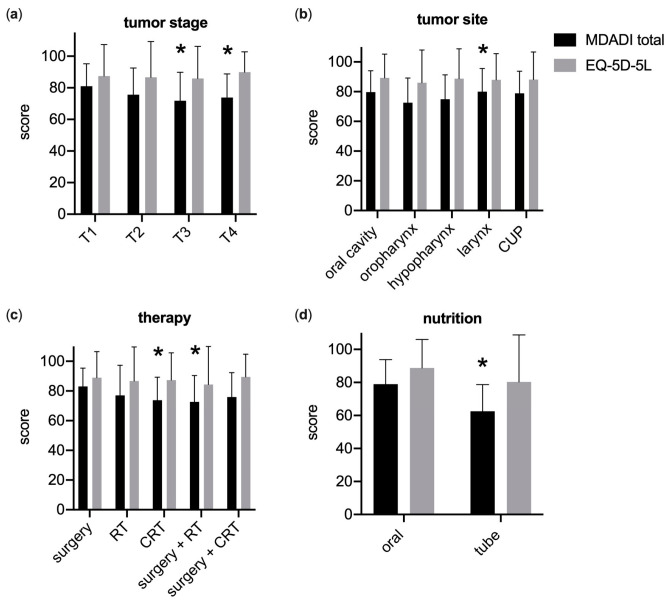
Swallowing-related QoL (MDADI total score) deteriorates in advanced head and neck cancer, while generic QoL (EQ-5D-5L) remains unrelated to cancer T-stage. (**a**) Low MDADI scores were associated with increasing cancer T-stage (* *p* < 0.05 vs. T1). (**b**) Cancer of the oral cavity, of the larynx, and unknown primary (CUP) were associated with high MDADI scores. Oropharyngeal and hypopharyngeal cancer scored a decrease (* *p* < 0.05 vs. oropharynx). (**c**) Among all patients and tumor locations, the MDADI score was highest for surgery only and significantly lower for combination therapies such as surgery + radiotherapy (RT) and chemoradiation (CRT) * *p* < 0.05 vs. surgery). (**d**) Feeding tube dependence scored a significant decrease for the MDADI (* *p* < 0.05 vs. oral nutrition). (**a**–**c**) However, generic quality of life did not change for increased cancer stage, cancer location, or therapy (*p* < 0.05). *n* = 59–112. Mean ± SD, (**a**–**c**) two-way ANOVA, Tukey; (**d**) Welch’s *t*-test.

This study’s analysis of general quality of life (QoL) through the EQ-5D-5L index suggested minimal variation across HNSCC patient groups, with consistently high scores averaging 0.88 ± 0.20 (Figure 1). In patients with T1 glottic laryngeal carcinoma, where minimal impact on quality of life is expected, the EQ-5D-5L score was found to be the same, at 0.89 ± 0.19. Notably, a significant difference emerged when comparing patients with oral nutrition to those with feeding tubes, indicating better QoL for the former group (0.89 ± 0.17 vs. 0.80 ± 0.28, *p* < 0.05). No significant differences were found when QoL was analyzed against tumor size, treatment method, tumor location, HPV status, or nutritional status.

In contrast, swallowing-related QoL, measured by the MDADI, was notably affected in severe HNSCC. Patients with T1 tumors reported the highest swallowing-related QoL (MDADI total score of 81.03 ± 14.20), while scores decreased in T3 (71.86 ± 17.99) and T4 (73.88 ± 14.94) tumors, indicating a statistically significant difference (*p* < 0.05). The highest scores were recorded for T1 glottic laryngeal carcinomas, with an average of 83.82 ± 11.60.

However, this disparity did not meet the clinically significant threshold of a 10-point difference. Surgery as a singular treatment modality was associated with better swallowing-related QoL than combined surgery + radiotherapy and primary radio(chemo)therapy, with significant and clinically meaningful differences noted.

QoL was also superior in patients who maintained oral nutrition (79.00 ± 14.82) as opposed to those dependent on tube feeding (62.55 ± 16.17), with both statistically and clinically significant findings (*p* < 0.05, r = 0.33). Tumor location influenced swallowing-related QoL, with the highest scores observed in patients with laryngeal (80.02 ± 15.59) and oral cavity cancer (79.71 ± 14.40). MDADI was lowest in oropharyngeal (72.60 ± 16.57; *p* < 0.05 vs. larynx) and hypopharyngeal cancers (74.87 ± 16.40).

Interestingly, HPV status showed no difference in swallowing-related QoL among patients with oropharyngeal cancer (72.58 ± 16.25 and 71.50 ± 16.16; *p* = 0.48). Additionally, treatment modality did not significantly affect MDADI scores in oropharyngeal tumors. When comparing treatment options, primary surgery + (chemo)radiotherapy yielded 73.31 ± 17, while primary (chemo)radiotherapy resulted in 71.45 ± 16. This similarity extended to MDADI subscales, the composite score, and global scores. For the time elapsed after therapy, a longer duration was associated with improvements in swallowing-related quality of life (QoL), with the association being statistically significant (*p* < 0.05). Patients surveyed more than five years after their treatment reported notably better scores (79.95 ± 14.43) compared with those surveyed less than six months post-therapy (70.86 ± 13.51, *p* < 0.05) and those surveyed between six to twelve months post-therapy (71.39 ± 15.29, *p* < 0.05). However, the statistical strength of this effect was relatively weak (f = 0.20). The Pearson correlation also revealed a weak positive correlation between time after treatment and the total MDADI score (r = 0.13, *p* < 0.05).

### 3.3. Fiberoptic Endoscopic Evaluation of Swallowing

**Figure 2:**
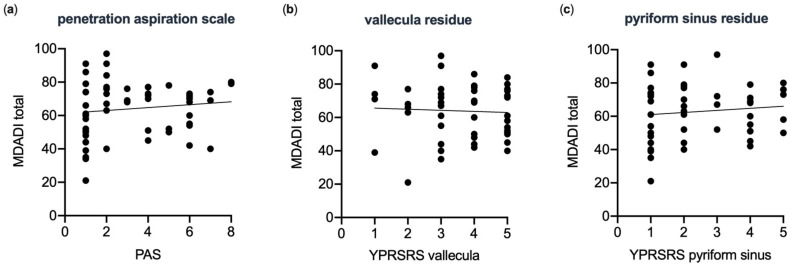
The correlation between the subjective swallowing-related quality of life (MDADI) and the objective fiber endoscopic evaluation of swallowing (FEES) is weak in head and neck cancer patients. No significant correlation was found between (**a**) MDADI and Rosenbek Penetration–Aspiration Scale (PAS) (*n* = 59, Spearman r = 0.17, *p* = 0.2); (**b**) MDADI and Yale Pharyngeal Residue Severity Rating Scale (YPRSRS) vallecula residues (*n* = 49, Spearman r = −0.035, *p* = 0.8); (**c**) MDADI and YPRSRS pyriform sinus residues (*n* = 48, Spearman r = 0.12, *p* = 0.44).

The objective evaluation of swallowing function via FEES across the study cohort (*n* = 59) yielded a median PAS score of 2 (range 1 to 5.25). Residue in the valleculae, as measured by the YPRSRS, was scored at 4 (range 3/5), and residue in the pyriform sinus at 2 (range 1/4). There was no observed correlation between the MDADI and objective measures of swallowing function; Spearman’s ρ between the MDADI total score and PAS was 0.20 (*p* = 0.27) (Figure 2). Mean MDADI scores did not significantly differ between patients with and without aspiration as per FEES (64.79 ± 12.71 vs. 63.76 ± 17.08, respectively; *p* = 0.94), nor did YPRSRS correlate with MDADI (valleculae residue: r = −0.05, *p* = 0.8; pyriform sinus residue: r = 0.12, *p* = 0.44).

**Figure 3:**
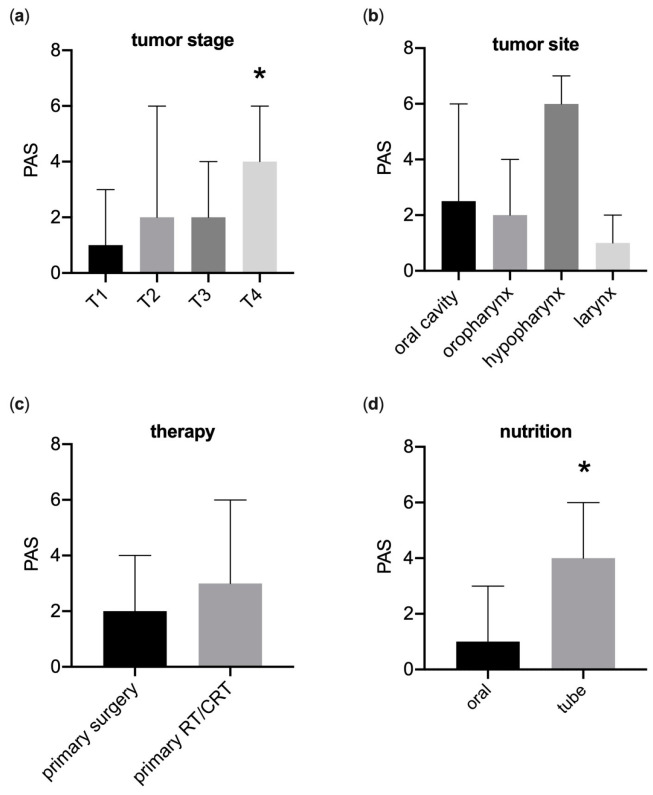
Fiberendoscopic swallow examination demonstrates a high specificity in identifying severely affected patients. (**a**) High Rosenbeks Penetration–Aspiration scale (PAS) scores were associated with advanced cancer T-stages (* *p* < 0.05 vs. T1). (**b**) PAS scores were comparable in patients after primary surgery and primary radiation (RT) or chemoradiation (CRT) (*p* = 0.15). (**c**) Impact of tumor location on penetration and aspiration scale (*p* = 0.10). (**d**) Association between feeding tube dependence on penetration and aspiration scale (* *p* < 0.05). *n* = 5–25. Median (95% CI); (**a**,**c**) Kruskal–Wallis test, Dunn’s multiple comparisons; (**b**,**d**) Mann–Whitney-U test.

The analysis also revealed that larger tumors were associated with higher PAS scores, indicating more pronounced penetration or aspiration events (Figure 3). Stage T1 tumors had significantly lower PAS scores (median 1, range 1/2) compared to stage T4 tumors (median 4, range 2/6) (*p* < 0.05). Tumors classified as T2 and T3 showed a median PAS of 2 (range 1/5), with an effect size (r) of 0.4, suggesting a moderate effect. Patients receiving oral nutrition demonstrated significantly better PAS scores (median 1, range 1/3.5) compared to those on PEG feeding (*p* < 0.05). The distribution of PAS scores was not significantly influenced by tumor location (*p* = 0.29) or treatment modality (*p* = 0.26). The most significant swallowing impairments were noted in hypopharyngeal tumors (PAS 6, range 3.2/7), followed by tumors in the oral cavity (median PAS 2.5, range 1/5.3), oropharynx (median PAS 2, range 1/4.5), and larynx (median PAS 1, range 1/2). Moreover, the type of therapy administered did not significantly affect penetration or aspiration rates (*p* = 0.26). However, a slight correlation was found with primary surgery showing a lower median PAS than definitive radio(chemo)therapy (median 2, range 1/4 vs. median 3, range 1/6), although this was not statistically significant.

### 3.4. Completion Rates of Digital Questionnaires in Cancer Aftercare

Regarding adopting digital technology for completing the questionnaires, of the 204 patients who attempted, 79% (*n* = 161) did so independently, and 21% (*n* = 43) needed help. Among those who completed it independently, 88% (*n* = 143) answered all questions. The completion rate for digital questionnaires (88%) was similar to the completion rate for paper-based questionnaires (82%, *n* = 75), with no significant difference in completion rates (*p* = 0.25). The ability to use the tablet decreased with age, with a 91% proficiency rate (*n* = 11 out of 12) for those under 50 years, 83% (*n* = 35 out of 42) for those aged 50 to 59 years, 85% (*n* = 75 out of 88) for the 60 to 69 age group, 68% (*n* = 27 out of 40) for those aged 70 to 79 years, and 59% (*n* = 13 out of 22) for those over 80 years old, showing a significant correlation with age (*p* < 0.05, R^2^ = 0.076).

## 4. Discussion

The present study investigated the correlation between subjective swallowing-related QoL and objective swallowing function in HNSCC. While the swallowing-related QoL correlated with the severity of the tumor disease, generic QoL remained unchanged in advanced disease. Notably, this study revealed an absence of correlation between objective swallowing measures (PAS; vallecula or piriform sinus residue) and patients’ perception of their level of pathophysiology using questionnaires. Due to this absence of correlation, in studies and clinical practice, the swallowing function should be assessed objectively alongside the patients’ subjective perception of swallowing.

In this context, generic QoL does not provide insight into the severity of head and neck cancer nor the functional success of the therapy. The generic QoL scores were relatively elevated across the entire group (0.88 ± 0.20). T1 glottic laryngeal carcinoma patients also demonstrated stable EQ-5D-5L values at 0.89 ± 0.19. Given the low disease burden and the effects of therapy, this group is not expected to experience a considerable impact on generic and, specifically, swallowing-related quality of life, thereby serving as a potential internal control group with similar socioeconomic status, age, and risk factors. The scores exceeded the German elderly population norms (0.84 ± 0.22) recently reported by Marten et al., indicating that the EQ-5D-5L questions are unsuitable for the head and neck cancer study cohort [39]. Additionally, no substantial disparities were detected among the different groups. For example, the QoL for T1 tumors was 0.87 ± 0.20, and for T4 tumors, it was 0.89 ± 0.13 (*p* = 0.95). Only tube feeding had such a negative impact on the QoL that it even reached significance in the generic quality of life assessment. This disparity highlights the complex nature of patients’ experiences, where their perception of well-being may differ from measurable physiological indicators. It underscores the need to incorporate a more comprehensive and nuanced approach to evaluating treatment outcomes.

Swallowing-related QoL (such as MDADI) is better suited to assess the severity of tumor disease and therapy success. While generic QoL offers insights into overall well-being, swallowing-related QoL assessments provide a deeper understanding of the specific challenges faced by head and neck cancer patients, particularly about their ability to swallow and maintain proper nutrition. This specialized focus makes swallowing-related QoL a more appropriate and informative tool for assessing treatment success and tailoring interventions to improve patients’ functional outcomes and, therefore, the overall QoL. In contrast to generic QoL, extensive tumors, feeding tube dependency, and a short time after therapy were significantly associated with low swallowing-related QoL. This correlation becomes particularly evident when examining tumor size in terms of T-Stage. Patients with T1 tumors, and especially those with glottic T1 tumors, exhibited significantly better quality of life (QoL) scores compared to those with advanced tumors. Specifically, patients with T1 tumors reported the highest swallowing-related QoL, as reflected in the MDADI total score of 81.03 ± 14.20. In contrast, QoL scores for T3 and T4 tumors were lower, at 71.86 ± 17.99 and 73.88 ± 14.94, respectively (*p* < 0.05). Notably, the highest scores were observed in T1 glottic laryngeal carcinomas, which averaged 83.82 ± 11.60. Overall, the values presented in the MDADI were relatively high compared to the head and neck cancer patients validation cohort of the German MDADI (60.9 ± 25.7) [33]. The values, however, are only limitedly comparable as the validation study included only patients with oral cavity carcinoma, and these patients were significantly younger.

The therapy itself had a low impact on the swallowing-related QoL. In the oropharyngeal carcinoma group, <12 months after the treatment, a similar pattern emerged as in the Orator study, with higher swallowing-related QoL among primarily irradiated patients [11,12]. Beyond 12 months, the benefit diminished, with surgery patients experiencing better swallowing-related QoL. Thus, these data support the hypothesis that function and QoL are independent of the treatment decision, except when the therapy is transoral surgery only. This finding aligns with previous research reporting that surgery only in oropharyngeal cancer is beneficial and highlights the potential of reducing radiation doses in HNSCC [40,41,42,43]. However, it is important to consider that the stage of the tumor correlates with the escalation of therapy. Surgery-only is typically indicated for T1/T2 N0 stage tumors.

However, data regarding subjective swallowing function must be interpreted with caution; to gauge the extent of dysphagia, it is essential to measure objective parameters. For instance, the MDADI was similar for patients with and without aspiration (63.94 ± 12.86; 64.02 ± 17.33, *p* = 0.20). Thus, the MDADI may not reliably identify severely affected patients with a potentially dangerous swallowing disorder. An objective examination, such as FEES, was essential to identify patients with severe impairments, such as silent aspiration. This emphasizes the need for a more robust evaluation that considers objective indicators. Incorporating both patient-reported outcomes and objective parameters in dysphagia assessment allows for a more comprehensive and nuanced approach to understanding the condition. While self-assessment tools provide valuable insights into the patient’s perspective, objective measures offer crucial clinical information that can guide treatment decisions, interventions, and rehabilitation strategies. This holistic approach enables patients to receive appropriate care that addresses both their individual experiences and the underlying physiological challenges they may be facing.

Several factors can diminish the validity of patient-reported outcome measures in HNSCC. Nerve injuries, tracheotomies, and radiotherapy can alter the subjective perception of dysphagia due to sensory loss [44]. This sensory loss can lead to a cessation of protective reflexes, thereby promoting silent aspirations. The incidence of silent aspirations is estimated to be 50–75% [44,45,46,47]. Additionally, QoL is a multifactorial construct: Swallowing-related QoL may be affected by dysphagia and other symptoms of oral dysfunction, such as dry mouth or loss of taste. Patients with oral cavity carcinoma and depression reported poor swallowing-related QoL in the MDADI [48]. Statements such as “I take longer to eat because of my swallowing problem” or “I feel like I have to swallow large amounts at once” can also be influenced by the presence of dry mouth. For patients, aspiration, penetration, or residue may be less restrictive to swallowing-related QoL than a disruption in the oral phase. This is supported by the finding that patients with oral cavity and oropharyngeal cancer, which frequently involve oral phase dysfunction, reported significantly worse swallowing-related QoL than patients with laryngeal or hypopharyngeal cancer [48]. In the present study, however, swallowing-related QoL was better in oral cavity cancer than in oro- and hypopharyngeal cancer. This discrepancy can be explained by the heterogeneity in the respective experimental groups, with more advanced hypopharyngeal than oral cavity cancer in this study (58% vs. 32%).

Previous research on the correlation between objective findings and QoL is highly controversial. In head and neck cancer patients, this limited correlation was observed when comparing multiple questionnaires alongside different accurate diagnostic methods in heterogeneous patient cohorts [18,19,20,21,22,23,24,25,26,27,28,29,49,50]. Standardized objective diagnostics, such as FEES [17,20,21,22,23,24] or VFSS [18,19,22,25,26,27,28,29], were commonly utilized, primarily focusing on measuring penetration and aspiration during swallowing. Among the questionnaires employed, the MD Anderson Dysphagia Inventory (MDADI) emerged as the most frequently used [17,19,20,22,23,26,27,34]. Further less often used questionnaires are Eating Assessment Tool (Eat-10) [18], Head and Neck 35 (H&N35) [21,28], and study-specific questionnaires [24,29]. Florie et al. found a lack of correlation using the Dutch MDADI and FEES in a cross-sectional design. Pedersen et al. used a prospective design to confirm the weak correlation between MDADI and FEES. Despite the overall weak correlation between the MDADI scores and objective diagnostic results in severely affected patients, statistically significant and occasionally clinically relevant differences of 10 points were observed by other studies: A substantial study on mainly oropharyngeal squamous cell carcinoma (OPSCC) (HPV Status not given) by Hutcheson et al. found associations between dysphagia in VFSS and the MDADI. A difference of 10 points in the MDADI composite was considered clinically significant [34]. Wishart et al. revealed significant correlations between objective measurements of pharyngeal swallow physiology and swallowing-related QoL [27]. These findings suggest a more robust concordance between VFSS and MDADI, primarily during the first three months after (C)RT. Hedström (FEES) and Pauloski (VFSS) found similar moderate correlations using study-specific questionnaires. The patient cohort was primarily composed of HPV-positive cases (88%) [15,24]. Studies that have identified a correlation between objective and subjective results are mainly from the Anglo–American region and included a high proportion of OPSCC cases. Overall, up to 88% were HPV-associated. However, Kendall et al. found a remarkable lack of correlation in a similar group of OPSCC patients [26]. The current understanding of the agreement between clinician-rated and patient-reported measures of dysphagia in HNSCC is still limited. The inconsistencies in study findings are attributed to several factors: sample sizes, cross-sectional analyses used in most studies, and population variables, including the heterogeneity in HPV positivity, treatment modalities, the severity of presenting symptoms, and the timing of data collection post-treatment. The present study further enhances the evidence that subjective QoL is a poor indicator of swallowing function, especially in predominantly HPV-negative patient populations.

In the discourse surrounding the adequacy of collecting subjective parameters related to swallowing function, the current study’s findings illustrate that disease-related QoL is considerably more appropriate than generic QoL. Nevertheless, this study further proves that disease-specific QoL, as observed in the ORATOR study, falls short of adequately assessing outcomes in everyday practice and clinical investigations.

The assessment of PROs in clinical studies and everyday practice should complement the objective measurement of swallowing function. Capturing disease-specific QoL is valid for eliciting the patient’s perspective on the issue of functional impairment. However, a comprehensive evaluation of the therapeutic success and determining the need for intervention requires the inclusion of objective functional assessments. This approach is essential to detect dangerous complications, such as silent aspiration. FEES and VFSS are the established methods of choice for this purpose. However, both methods have the drawback of being time-consuming and resource-intensive, leading to their utilization in only 30% of studies focusing on swallowing function [13]. In the present study, FEES was chosen as the standard examination due to its ability to allow multiple assessments without radiation exposure and its direct visualization of laryngeal function, including aspiration and penetration. FEES facilitates the visualization of aspiration and allows for assessing airway protection mechanisms. However, its capabilities have significant limitations. The evaluation of the oral phase of swallowing is constrained. The visualization of the larynx during the pharyngeal phase is interrupted due to the transient ‘white out’ effect, which occurs when the soft palate elevates and pharyngeal constriction occurs. In instances of intradeglutitive aspiration, the quantity of aspirated material can only be estimated. Factors such as secretions and post-therapeutic alterations in anatomy can impair visibility. Additionally, FEES does not provide the ability to assess disturbances in the esophageal phase of swallowing.

Conversely, VFSS offers dynamic imaging that extends from the oral to the esophageal phases. This modality provides comprehensive insights into the movement of all pertinent structures and the bolus, including the precise quantification of aspirated material. However, it has drawbacks; VFSS necessitates the use of radiation and contrast medium, which can alter the viscosity of the bolus. Moreover, research indicates that VFSS tends to systematically underrate the presence of residue and penetration/aspiration events.

Despite these drawbacks, VFSS is exceptionally suited for specific inquiries concerning swallowing functions. It is essential to recognize that FEES and VFSS are complementary diagnostic tools for a comprehensive evaluation of swallowing function.

Regarding skills in handling information technology, the patient cohort performed well. The acceptance and proficiency in managing tablet-based questionnaires were high within the HNSCC patient group. A total of 79% of the patients could independently complete the questionnaires, and the level of completeness was comparable with that of analog questionnaires. The ability to engage with digital questionnaires was significantly associated with age. The age group of 60 to 69 years, which also exhibited the highest incidence of HNSCC in this study and the general population, demonstrated an 85% capability in independently completing the digital questionnaire. The findings support the implementation of digital questionnaires with automated analysis, enabling the sustainable collection of PROs.

This study encompasses a heterogeneous cross-section of Central European head and neck cancer patients: This patient cohort comprised various age groups, tumors from diverse locations, and a broad post-treatment period. The patients differ significantly from previous studies due to a high proportion of cancer attributable to smoking and/or alcohol (-non-HPV) in advanced stages. This group of patients substantially diverges from the typical Anglo–American study subjects and necessitates supplementary evaluation. Therefore, the examined patients provide an overview of this often-underrepresented clientele. Notable parallels are evident with Dutch patients, where only a modest association between H&N35 and VFSS could be established [28]. However, an actual swallowing-related questionnaire was yet to be available in Dutch at the time of the study. Recently, the MDADI has established itself as the most widely used QoL questionnaire for head and neck tumor patients in clinical studies (ORATOR, E3311, Pathos, HN002) [12,30,31,32]. Hence, the examination of an evaluated and translated MDADI was imperative. 

This study design is primarily suited to provide an overview of HNSCC patients. The heterogeneous etiology, varying treatments, and a limited number of patients with MDADI and FEES made sufficient stratification into etiological and treatment groups unfeasible. Moreover, each patient was assessed only at a single time point. Although this approach demonstrated minimal changes in functional impairment after one year, the repeated evaluation of the same patients, especially regarding MDADI, could have eliminated patient-specific variations. This limitation partly explains the detection of <10 points differences in MDADI, particularly given that MDADI was designed as a prospective study component [34]. Therefore, the transferability of these data to other patient groups and different diseases is limited due to the heterogeneity in the study participants and the design as a cross-sectional study (including lack of causality, time-dependent biases, and cohort effects).

A significant limitation of this study is its exclusive reliance on FEES. The predominance of previous research utilizing VFSS affects comparability due to its advanced capabilities in analyzing bolus movement and detailed structure assessment. However, FEES was selected for this study’s swallowing assessments for its thorough anatomical evaluation of the pharynx and larynx. FEES is a recognized, safe (radiation-free), and commonly used diagnostic tool in the management and therapeutic planning of swallowing disorders [51]. It enables frequent re-evaluations with minimal patient risk, proving particularly beneficial for tailoring therapeutic interventions to meet oncological treatment patients’ evolving needs and symptoms.

Despite these advantages, it’s important to note that FEES may underrepresent challenges encountered during the oral swallowing phase, which can significantly impact the patient’s quality of life. Therefore, a comprehensive clinical assessment of the oral phase remains essential to understand and address swallowing difficulties fully.

The study results have practical implications, indicating that an objective assessment is essential for each swallowing evaluation in clinical studies. We recommend FEES due to its repetitive applicability and ability to identify hazardous complications reliably. Through standardized execution, according to Langmore, the amount of residue and risk of penetration/aspiration can be examined for different consistencies [38]. 

To accommodate limited resources, an initial screening assessment can be carried out. The 100 mL swallowing test [52,53] and the Water Swallowing Test (WST) [54] exhibit high sensitivity and specificity in diagnosing dysphagia. In the presence of abnormal findings, an indication for further assessment using FEES or VFSS is warranted. However, the screening tests above possess limited predictive ability for detecting (silent) aspirations due to sensory impairments following treatment. 

Silent aspiration is also a limitation for the revision of questionnaires to identify potentially dangerous complications. The commonly used swallowing-related QoL questionnaires include specific questions about aspiration. For instance, the MD Anderson Dysphagia Inventory (MDADI) asks, ‘I cough when I try to drink liquids,’ and the recently updated extensive EORTC QLQ–H&N43 includes questions such as ‘Have you had problems with coughing?’ and ‘Have you had problems swallowing liquids?’. These questions could potentially be refined to more precisely inquire about dangerous complications such as aspiration, or they could be given greater weight in the analysis. However, this approach does not address the critical issue of silent aspiration. Questionnaires are limited in their length and cannot effectively capture complications that are not noticed by patients. 

Further research should confirm the findings in a multicentric fashion on stratified patients (based on localization, therapy, and HPV status). Additionally, future investigations should employ a prospective design with different evaluation time points (e.g., pre-therapeutic, at 3-, 6-, and 12-months post-therapy) to account for the slight differences in MDADI scores and find specific functional problems during the post-therapy stages. Furthermore, research on MDADI should examine whether more minor differences than 10 points hold clinical significance.

## 5. Conclusions

This study’s results indicate that patient-rated questionnaires, such as the MDADI, can provide valuable insights into assessing swallowing-related QoL among patients with Head and Neck Squamous Cell Carcinoma (HNSCC). However, these findings do not correspond with objective measures of swallowing function. Relying solely on PROs leads to omitting crucial aspects of swallowing function, which needs to be improved in clinical studies, registries, and everyday situations. This lack of correlation underscores the significance of adopting a comprehensive approach that considers both PROs and objective evaluations, such as FEES or VFSS.

## Figures and Tables

**Table 1:** Characteristics of the Study Cohort.

Group Characteristics	EQ-5D-5L, MDADI *n* = 307	FEES, EQ-5D-5L, MDADI *n* = 59
Age [years]	65.88 (±9.98)	63.20 (±9.32)
Female	62 (20.33%)	13 (22.03%)
Male	245 (79.81%)	46 (77.97%)
Primary tumor T size		
T1	92 (29.97%)	11 (18.64%)
T2	81 (26.38%)	11 (18.64%)
T3	56 (18.24%)	17 (28.81%)
T4	56 (18.24%)	19 (32.20%)
Tx	22 (7.21%)	1 (1.69%)
Primary tumor location		
Oral cavity	24 (7.82%)	10 (16.95%)
Nasopharynx	4 (1.30%)	0
Oropharynx	124 (40.39%)	33 (55.93%)
Hypopharynx	30 (9.77%)	6 (10.17%)
Larynx	99 (32.25%)	9 (15.25%)
CUP	22 (7.17%)	1 (1.69%)
Other	4 (1.30%)	0
Therapy		
Surgery only	74 (24.10%)	9 (15.25%)
(Chemo)Radiotherapy	113 (36.72%)	26 (44.06%)
Surgery + Radiotherapy	58 (18.89%)	13 (22.03%)
Surgery + Chemoradiotherapy	61 (19.87%)	11 (18.64%)
Time after therapy		
≤5 months	32 (10.42%)	28 (47.46%)
6–11 months	41 (13.36%)	12 (20.34%)
1–5 years	141 (45.93%)	15 (25.41%)
>5 years	93 (30.29%)	4 (6.78%)
Feeding tube use	47 (15.31%)	33 (55.93%)

## Data Availability

The data presented in this study are available on request from the corresponding author.

## References

[1] Malone E., Siu L.L. (2018). Precision Medicine in Head and Neck Cancer: Myth or Reality?. Clin. Med. Insights Oncol..

[2] Guo T., Califano J.A. (2015). Molecular Biology and Immunology of Head and Neck Cancer. Surg. Oncol. Clin. N. Am..

[3] Specenier P., Vermorken J.B. (2018). Optimizing Treatments for Recurrent or Metastatic Head and Neck Squamous Cell Carcinoma. Expert Rev. Anticancer Ther..

[4] Billard-Sandu C., Tao Y.G., Sablin M.P., Dumitrescu G., Billard D., Deutsch E. (2020). CDK4/6 Inhibitors in P16/HPV16-Negative Squamous Cell Carcinoma of the Head and Neck. Eur. Arch. Otorhinolaryngol..

[5] Ordoñez R., Otero A., Jerez I., Medina J.A., Lupiañez-Pérez Y., Gomez-Millan J. (2019). Role of Radiotherapy in the Treatment of Metastatic Head and Neck Cancer. OncoTargets Ther..

[6] Hutcheson K.A., Nurgalieva Z., Zhao H., Gunn G.B., Giordano S.H., Bhayani M.K., Lewin J.S., Lewis C.M. (2019). Two-Year Prevalence of Dysphagia and Related Outcomes in Head and Neck Cancer Survivors: An Updated SEER-Medicare Analysis. Head Neck.

[7] Eisbruch A. (2004). Dysphagia and Aspiration Following Chemo-Irradiation of Head and Neck Cancer: Major Obstacles to Intensification of Therapy. Ann. Oncol..

[8] Nguyen N.P., Frank C., Moltz C.C., Vos P., Smith H.J., Bhamidipati P.V., Karlsson U., Nguyen P.D., Alfieri A., Nguyen L.M. (2006). Aspiration Rate Following Chemoradiation for Head and Neck Cancer: An Underreported Occurrence. Radiother. Oncol..

[9] Nguyen N.P., Moltz C.C., Frank C., Vos P., Smith H.J., Karlsson U., Dutta S., Midyett F.A., Barloon J., Sallah S. (2004). Dysphagia Following Chemoradiation for Locally Advanced Head and Neck Cancer. Ann. Oncol..

[10] Abdelhafiz N., Mahmoud D., Gad M., Essa H., Morsy A. (2023). Effect of Definitive Hypo-Fractionated Radiotherapy Concurrent with Weekly Cisplatin in Locally Advanced Squamous Cell Carcinoma of the Head and Neck. J. Med. Life.

[11] Nichols A.C., Theurer J., Prisman E., Read N., Berthelet E., Tran E., Fung K., de Almeida J.R., Bayley A., Goldstein D.P. (2022). Randomized Trial of Radiotherapy Versus Transoral Robotic Surgery for Oropharyngeal Squamous Cell Carcinoma: Long-Term Results of the ORATOR Trial. J. Clin. Oncol..

[12] Nichols A.C., Theurer J., Prisman E., Read N., Berthelet E., Tran E., Fung K., de Almeida J.R., Bayley A., Goldstein D.P. (2019). Radiotherapy versus Transoral Robotic Surgery and Neck Dissection for Oropharyngeal Squamous Cell Carcinoma (ORATOR): An Open-Label, Phase 2, Randomised Trial. Lancet Oncol..

[13] Li P., Constantinescu G.C., Nguyen N.T.A., Jeffery C.C. (2020). Trends in Reporting of Swallowing Outcomes in Oropharyngeal Cancer Studies: A Systematic Review. Dysphagia.

[14] Campbell B.H., Spinelli K., Marbella A.M., Myers K.B., Kuhn J.C., Layde P.M. (2004). Aspiration, Weight Loss, and Quality of Life in Head and Neck Cancer Survivors. Arch. Otolaryngol. Head Neck Surg..

[15] Pauloski B.R., Rademaker A.W., Logemann J.A., Lazarus C.L., Newman L., Hamner A., MacCracken E., Gaziano J., Stachowiak L. (2002). Swallow Function and Perception of Dysphagia in Patients with Head and Neck Cancer. Head Neck.

[16] Agarwal J., Palwe V., Dutta D., Gupta T., Laskar S.G., Budrukkar A., Murthy V., Chaturvedi P., Pai P., Chaukar D. (2011). Objective Assessment of Swallowing Function After Definitive Concurrent (Chemo)Radiotherapy in Patients with Head and Neck Cancer. Dysphagia.

[17] Florie M., Pilz W., Kremer B., Verhees F., Waltman G., Winkens B., Winter N., Baijens L. (2021). EAT-10 Scores and Fiberoptic Endoscopic Evaluation of Swallowing in Head and Neck Cancer Patients. Laryngoscope.

[18] Arrese L.C., Carrau R., Plowman E.K. (2017). Relationship Between the Eating Assessment Tool-10 and Objective Clinical Ratings of Swallowing Function in Individuals with Head and Neck Cancer. Dysphagia.

[19] Gillespie M.B., Brodsky M.B., Day T.A., Lee F.-S., Martin-Harris B. (2004). Swallowing-Related Quality of Life After Head and Neck Cancer Treatment. Laryngoscope.

[20] Pedersen A., Wilson J., McColl E., Carding P., Patterson J. (2016). Swallowing Outcome Measures in Head and Neck Cancer—How Do They Compare?. Oral Oncol..

[21] Jensen K., Lambertsen K., Torkov P., Dahl M., Bonde Jensen A., Grau C. (2007). Patient Assessed Symptoms Are Poor Predictors of Objective Findings. Results from a Cross Sectional Study in Patients Treated with Radiotherapy for Pharyngeal Cancer. Acta Oncol..

[22] Kirsh E., Naunheim M., Holman A., Kammer R., Varvares M., Goldsmith T. (2019). Patient-reported versus Physiologic Swallowing Outcomes in Patients with Head and Neck Cancer after Chemoradiation. Laryngoscope.

[23] da Silva G.M., Portas J., López R.V.M., Côrrea D.F., Arantes L.M.R.B., Carvalho A.L. (2019). Study of Dysphagia in Patients with Advanced Oropharyngeal Cancer Subjected to an Organ Preservation Protocol Based on Concomitant Radiotherapy and Chemotherapy. Asian Pac. J. Cancer Prev..

[24] Hedström J., Tuomi L., Finizia C., Olsson C. (2018). Correlations Between Patient-Reported Dysphagia Screening and Penetration–Aspiration Scores in Head and Neck Cancer Patients Post-Oncological Treatment. Dysphagia.

[25] Liou H.-H., Tsai S.-W., Hsieh M.H.-C., Chen Y.-J., Hsiao J.-R., Huang C.-C., Ou C.-Y., Chang C.-C., Lee W.-T., Tsai S.-T. (2022). Evaluation of Objective and Subjective Swallowing Outcomes in Patients with Dysphagia Treated for Head and Neck Cancer. J. Clin. Med..

[26] Kendall K.A., Kosek S.R., Tanner K. (2014). Quality-of-life Scores Compared to Objective Measures of Swallowing after Oropharyngeal Chemoradiation. Laryngoscope.

[27] Wishart L.R., Harris G.B., Cassim N., Alimin S., Liao T., Brown B., Ward E.C., Nund R.L. (2022). Association Between Objective Ratings of Swallowing and Dysphagia-Specific Quality of Life in Patients Receiving (Chemo)Radiotherapy for Oropharyngeal Cancer. Dysphagia.

[28] van der Molen L., van Rossum M.A., Ackerstaff A.H., Smeele L.E., Rasch C.R., Hilgers F.J. (2009). Pretreatment Organ Function in Patients with Advanced Head and Neck Cancer: Clinical Outcome Measures and Patients’ Views. BMC Ear Nose Throat Disord..

[29] Rogus-Pulia N.M., Pierce M.C., Mittal B.B., Zecker S.G., Logemann J.A. (2014). Changes in Swallowing Physiology and Patient Perception of Swallowing Function Following Chemoradiation for Head and Neck Cancer. Dysphagia.

[30] Ferris R.L., Flamand Y., Weinstein G.S., Li S., Quon H., Mehra R., Garcia J.J., Chung C.H., Gillison M.L., Duvvuri U. (2022). Phase II Randomized Trial of Transoral Surgery and Low-Dose Intensity Modulated Radiation Therapy in Resectable P16+ Locally Advanced Oropharynx Cancer: An ECOG-ACRIN Cancer Research Group Trial (E3311). J. Clin. Oncol..

[31] Owadally W., Hurt C., Timmins H., Parsons E., Townsend S., Patterson J., Hutcheson K., Powell N., Beasley M., Palaniappan N. (2015). PATHOS: A Phase II/III Trial of Risk-Stratified, Reduced Intensity Adjuvant Treatment in Patients Undergoing Transoral Surgery for Human Papillomavirus (HPV) Positive Oropharyngeal Cancer. BMC Cancer.

[32] Yom S.S., Torres-Saavedra P., Caudell J.J., Waldron J.N., Gillison M.L., Xia P., Truong M.T., Kong C., Jordan R., Subramaniam R.M. (2021). Reduced-Dose Radiation Therapy for HPV-Associated Oropharyngeal Carcinoma (NRG Oncology HN002). J. Clin. Oncol..

[33] Bauer F., Seiss M., Gräßel E., Stelzle F., Klotz M., Rosanowski F. (2010). Schluckbezogene Lebensqualität Bei Mundhöhlenkarzinomen: Anderson-Dysphagia-Inventory, Deutsche Version. HNO.

[34] Hutcheson K.A., Barrow M.P., Lisec A., Barringer D.A., Gries K., Lewin J.S. (2016). What Is a Clinically Relevant Difference in MDADI Scores between Groups of Head and Neck Cancer Patients?. Laryngoscope.

[35] Rosenbek J.C., Robbins J.A., Roecker E.B., Coyle J.L., Wood J.L. (1996). A Penetration-Aspiration Scale. Dysphagia.

[36] Hey C., Pluschinski P., Zaretsky Y., Almahameed A., Hirth D., Vaerst B., Wagenblast J., Stöver T. (2014). Penetration-Aspiration Scale According to Rosenbek: Validation of the German Version for Endoscopic Dysphagia Diagnostics. HNO.

[37] Neubauer P.D., Rademaker A.W., Leder S.B. (2015). The Yale Pharyngeal Residue Severity Rating Scale: An Anatomically Defined and Image-Based Tool. Dysphagia.

[38] Langmore S.E., Kenneth S.M.A., Olsen N. (1988). Fiberoptic Endoscopic Examination of Swallowing Safety: A New Procedure. Dysphagia.

[39] Marten O., Greiner W. (2021). EQ-5D-5L Reference Values for the German General Elderly Population. Health Qual. Life Outcomes.

[40] Amit M., Hutcheson K., Zaveri J., Lewin J., Kupferman M.E., Hessel A.C., Goepfert R.P., Brandon Gunn G., Garden A.S., Ferraratto R. (2019). Patient-Reported Outcomes of Symptom Burden in Patients Receiving Surgical or Nonsurgical Treatment for Low-Intermediate Risk Oropharyngeal Squamous Cell Carcinoma: A Comparative Analysis of a Prospective Registry. Oral Oncol..

[41] Barbon C.E.A., Yao C.M.K.L., Alvarez C.P., Goepfert R.P., Fuller C.D., Lai S.Y., Gross N.D., Hutcheson K.A. (2021). Dysphagia Profiles after Primary Transoral Robotic Surgery or Radiation for Oropharyngeal Cancer: A Registry Analysis. Head Neck.

[42] Dohopolski M.J., Diao K., Hutcheson K.A., Akhave N.S., Goepfert R.P., He W., Lei X.J., Peterson S.K., Shen Y., Sumer B.D. (2023). Long-Term Patient-Reported Outcomes in a Population-Based Cohort Following Radiotherapy vs Surgery for Oropharyngeal Cancer. JAMA Otolaryngol. Head Neck Surg..

[43] Choby G.W., Kim J., Ling D.C., Abberbock S., Mandal R., Kim S., Ferris R.L., Duvvuri U. (2015). Transoral Robotic Surgery Alone for Oropharyngeal Cancer: Quality-of-Life Outcomes. JAMA Otolaryngol. Head Neck Surg..

[44] Strojan P., Hutcheson K.A., Eisbruch A., Beitler J.J., Langendijk J.A., Lee A.W.M., Corry J., Mendenhall W.M., Smee R., Rinaldo A. (2017). Treatment of Late Sequelae after Radiotherapy for Head and Neck Cancer. Cancer Treat. Rev..

[45] Feng F.Y., Kim H.M., Lyden T.H., Haxer M.J., Worden F.P., Feng M., Moyer J.S., Prince M.E., Carey T.E., Wolf G.T. (2010). Intensity-Modulated Chemoradiotherapy Aiming to Reduce Dysphagia in Patients With Oropharyngeal Cancer: Clinical and Functional Results. J. Clin. Oncol..

[46] Jagtap M., Karnad M. (2019). Swallowing Skills and Aspiration Risk Following Treatment of Head and Neck Cancers. Indian J. Surg. Oncol..

[47] Langerman A., MacCracken E., Kasza K., Haraf D.J., Vokes E.E., Stenson K.M. (2007). Aspiration in Chemoradiated Patients with Head and Neck Cancer. Arch. Otolaryngol. Head Neck Surg..

[48] Chen S.C., Huang B.S., Hung T.M., Chang Y.L., Lin C.Y., Chung C.Y., Wu S.C. (2018). Swallowing Ability and Its Impact on Dysphagia-Specific Health-Related QOL in Oral Cavity Cancer Patients Post-Treatment. Eur. J. Oncol. Nurs..

[49] Weymuller E.A., Yueh B., Deleyiannis F.W.B., Kuntz A.L., Alsarraf R., Coltrera M.D. (2000). Quality of Life in Patients With Head and Neck Cancer. Arch. Otolaryngol. Head Neck Surg..

[50] Morton R.P. (2003). Studies in the Quality of Life of Head and Neck Cancer Patients: Results of a Two-Year Longitudinal Study and a Comparative Cross-Sectional Cross-Cultural Survey. Laryngoscope.

[51] Lehner U., Zaretsky E., Goeze A., Wermter L., Stuck B.A., Birk R., Neff A., Fischer I., Ghanaati S., Sader R. (2022). Prätherapeutische Dysphagie Bei Kopf-Hals-Tumor-Patienten. HNO.

[52] Patterson J.M., Hildreth A., McColl E., Carding P.N., Hamilton D., Wilson J.A. (2011). The Clinical Application of the 100 ML Water Swallow Test in Head and Neck Cancer. Oral Oncol..

[53] Watson L.J., Woodman S.H., Ganderton D., Hutcheson K.A., Pringle S., Patterson J.M. (2022). Development of the Remote 100 Ml Water Swallow Test versus Clinical Assessment in Patients with Head and Neck Cancer: Do They Agree?. Head Neck.

[54] Hey C., Goeze A., Sader R., Zaretsky E. (2023). FraMaDySc: Dysphagia Screening for Patients after Surgery for Head and Neck Cancer. Eur. Arch. Oto-Rhino-Laryngol..

